# Does Young Adults’ Neighborhood Environment Affect Their Depressive Mood? Insights from the 2019 Korean Community Health Survey

**DOI:** 10.3390/ijerph18031269

**Published:** 2021-01-31

**Authors:** Da-Hye Yim, Youngsang Kwon

**Affiliations:** 1 Department of Civil and Environmental Engineering, Seoul National University, Seoul 08826, Korea; dahye72@snu.ac.kr; 2 Smart City Research Center, Advanced Institute of Convergence Technology, Seoul National University, Suwon 16229, Korea

**Keywords:** neighborhood environment, depressive mood, young adults, Tobit model

## Abstract

The rates of depression among young adults have been increasing in high-income countries and have emerged as a social problem in South Koreans aged 19–34. However, the literature is unclear on whether the neighborhood environment that young adults live in affects the onset and severity of their depressive symptoms. This study analyzed data from the 2019 Korean Community Health Survey (KCHS) using the Tobit model to identify the effect of the neighborhood environment on young adults’ depressive moods. Controlling for other corresponding factors, young adults’ neighborhood environment satisfaction affected their depression, and natural environment satisfaction (32.5%), safety level satisfaction (31.0%), social overhead capital (SOC), environment satisfaction (30.2%), trust between neighbors satisfaction (20.1%), and public transportation environmental satisfaction (12.2%) affected young adults’ depressive moods. Of these, natural environment satisfaction (32.5%), safety level environment satisfaction (31.0%), and SOC environment satisfaction (30.2%) affected young adults’ depressive mood to a similar extent. This implies that many young adults in South Korea live in inadequate neighborhood conditions. This research contributes to the literature by identifying the specific environmental factors that affect young adults’ depressive moods.

## 1. Introduction

### 1.1. Background

Depressive mood and depressive symptoms are on the rise worldwide. The World Health Organization (WHO) has predicted that depression will represent the largest illness-related burden worldwide in the future [1]. Severe depression is a mental illness that causes cognitive and functional degradation and, in many cases, leads to suicide. Detecting depression in its early stages is an effective and essential task because treating late-stage depression is enormously costly at the national level and very painful for long-time sufferers at the individual level [2]. Some high-income countries, such as Great Britain and Germany, have responded to this serious situation by carrying out national-level projects to detect and prevent depression in its early stages [3,4].

Severe depression is related to suicide, and suicide is a leading cause of death among young people worldwide [5]. This indicates that depression is a huge mental health issue for young people. In the United States, youth aged 18–25 had much higher rates of depression than other age groups in 2017 [6]. Between 2001 and 2018, suicide and addiction were the leading causes of death for young adults aged 20–34 in the United Kingdom [7]. In Japan, suicide was the leading cause of death for people aged 15–34 in 2018 [8].

According to recent statistics of the Korean National Health Insurance Service (2019), 26.4% of South Koreans aged 20–39 have been diagnosed with depression. To respond to this widespread social problem, South Korean national government agencies have enacted the *Framework Act on Young Adults* in 2020, which aims to improve the quality of life for young adults in all areas. Simultaneously, local governments in South Korea have begun carrying out mental health support projects for young adults. Prior studies in the South Korean context have revealed that unemployment [9] and the high cost of housing [10,11] are the driving factors which cause depression in young adults. Other factors—such as the relationship between depression and living alone, young adults’ living habits, and their living conditions—have not yet been clarified in the literature.

Previous studies have indicated that depression is caused by individual temperament, genetic predisposition (i.e., family history), and one’s environment and external factors [12]. Given that one’s living situation and neighborhood environment are related to one’s mental health [13], previous studies have underlined how individuals’ neighborhood environments could increase their stress and damage their mental health. Neighborhood factors, such as individuals’ socioeconomic status, feelings of safety [14], access to facilities and amenities [15], a neighborhood’s social density [16] or a lack of green and open space [17], and individuals’ social capital [18] are all related to depression.

### 1.2. Purpose

Although the neighborhood environment could affect the young adults’ depressive mood, the extent and details of this influence—such as which aspects of their neighborhood environment adversely affect their depressive mood—are not yet clear. This study aims to clarify the extent and details of neighborhood environments’ influence on young adults’ depressive moods in South Korea.

## 2. Literature Review

### 2.1. Young Adults’ Mental Health

During young adulthood, people establish their identity as independent individuals independent of their parents [14,15]. Erikson has defined early adulthood as taking place around age 20–39 [19] and Arnett has described the time between age 18 and one’s mid-20s as “emerging adulthood” [20]. Others have defined young adults as people aged 20–39 in consideration of the kinds of political and emotional support available to them [16,21,22]. In short, young adulthood can be loosely defined as the period between one’s 20s and their first marriage. 

Development theory asserts that depressive mood in young adults can be explained by their long-term unemployment [23] and anxiety over their future and academic burdens [24]. Other research has found that housing problems could affect young adults’ depression and mental health [10]. While other studies have asserted that young adults’ extensive use of smartphones and overly sedentary behavior [25] and their social network service usage [26] may affect depression in young adults. As mentioned earlier, it is important to diagnose and treat early-stage depression to treat depression effectively. This is because 75% of sufferers of mild and severe depression experience symptoms before the age of 24 [27] and early detection, treatment, and management of these early symptoms can aid the effective management of depressive symptoms later in life.

### 2.2. Factors Affecting Young Adults’ Depressive Moods

The Diagnostic and Statistical Manual of Mental Disorders (DSM-5) defines depression as a mental illness when someone’s depressive mood and symptoms last more than two weeks. Unlike other mental illnesses, the genetic characteristics of depression are not clearly defined [28]. Both genetic and non-genetic factors have been found to cause depression, often interacting with one another in complex ways [29]. Previous studies have identified that women suffer from depression more than men [30,31], and living in low-income housing and having debts [32] increase peoples’ depressive moods. Studies from Western countries have found that living in a high-rise apartment building can have adverse effects on mental health [33]. Studies from South Korea indicate that people living alone are more likely to experience depression [34]. Other studies have indicated that married individuals tend to be less depressed [30,35]. Just as unemployment has been found to be a cause for depression among young adults [9,23], work-related stress has been found to cause depression in individuals of all age groups [36].

Individuals’ physical health and lifestyles can also cause depression. Physical exercise helps reduce depressive mood even without psychological effects such as the placebo effect [37]. Walking [37], aerobic exercise, and anaerobic exercise have all been shown to effectively reduce peoples’ depressive moods [38]. However, it is not clear whether other types of exercise effectively alleviate individuals’ depressive moods [39]. Researchers have also found that heavy drinking and alcohol addiction are highly correlated with depressive moods [40,41]. Stress has been found to cause depression because of the hormonal problems and imbalances related to serotonin and the body’s stress hormone system [42]. Thus, stress is understood as a powerful predictor of depression. Some researchers view daily stress as a better predictor of depression than stress from major life events [39].

Social support has been found to alleviate depressive moods [43]. Relationships and social support have different levels of importance in this regard depending on a depressed person’s age. For example, adults have consistent support from their family, spouses, and friends or acquaintances [44]. However, when college students have a bad relationship with their parents, they are often more depressed or likely to be depressed [45]. Support from family has been found to relieve symptoms of depression [46], as has religious activity [47] and continuous volunteer activity [48]. Leisure activities only relieve depressive moods when they involve physical activity [49].

### 2.3. Effect of Neighborhood Environment on Depressive Moods

Both neighborhoods’ natural environment and built environment impact individuals’ depressive moods, either through personal cognitive processes or causing activities. Several scholars have proposed theoretical models which describe how personal control, social support, and positive distraction (or restoration) in a neighborhood environment have mediating impacts on individuals’ mental health [9,42]. However, further research is needed to give empirical support to this model [13].

The presence of green areas and public spaces, a neighborhood’s traffic conditions and safety, and trust between neighbors have all been found to affect people’s depressive moods. A lack of green space has been found to cause depressive mood in several studies [17] and exposure to the natural environment has been found to help relieve depressive moods and reduce stress [50]. Communal space in neighborhood environments has been found to have a positive impact on relieving depressive mood by helping people develop strong social relationships [51]. Similarly, trust between neighbors has been found to help reduce people’s depressive moods [52]. In addition, building deterioration and fear of crime have been found to be highly related with severe depression [14], and traffic noise has been found to adversely affect people’s depressive moods [13,53].

Many studies have focused on elderly people’s perceptions of their neighborhood environment. The real crime rate of a given neighborhood environment and the perceived safety of that neighborhood have been shown to affect the elderly people’s depressive moods [54]. In addition, elderly people’s lack of physical ability has been found to reduce their access to exercise and amenities which might improve their mood [54,55]. In short, social capital, as a mediating factor, can reduces depressive mood [56]. 

### 2.4. This Study’s Relationship to the Literature

This study differs from previous research because it examines the influence of the neighborhood environment on young adults’ depressive moods. Because depressive moods have a social dimension or cause, the cause of depressive moods may be different among people of different age groups. Because comparatively few studies have examined the cause of depressive moods and the influence of the neighborhood environment on depressive moods in young adults, this study makes a novel contribution to the literature. This study also contributes to the literature because young adults have fewer physical limitations than the elderly and thus our findings imply that they can recognize and help identify the cognitive, rather than physical, effects and causes of depressive moods.

## 3. Materials and Methods

### 3.1. Research Contents

#### 3.1.1. Research Questions

This study’s research questions are as follows. First, do young adults’ perceptions of their neighborhood environment affect their depressive mood? We hypothesize that the answer to this question is yes. Second, in what ways do each of the following neighborhood factors—social overhead capital (SOC) environment, natural environment, safety level, trust between members of the neighborhood, and public transportation environment—affect young adults’ depressive moods?

#### 3.1.2. Research Subjects

In the existing published studies, young adults are identified as those between 15 and 39 years [14,15,16,19,20,21,22]. For this study, we set the age range of young adults between 19 and 34 based on recent research trends and Korean law.

#### 3.1.3. Research Method

To determine which factors affect South Korean young adults’ depressive moods, we (1) compared the coefficients of young adult model (19–34) with the general adult model (35+) and (2) identified the impact of the neighborhood environment on Korean young adults’ depressive mood. 

We determined whether young adults’ satisfaction with their neighborhood environment affects their depressive mood by controlling for other factors (e.g., demographic and socioeconomic factors, social activities, physical activities, etc.). We analyzed whether satisfaction in each of these neighborhood environment variable influences young adults’ depressive moods.

We used national-level data from the 2019 Korean Community Health Survey (KCHS), and the Tobit model, which is appropriate for continuous dependent variables with truncated values. Although previous research has used multilevel models to identify the effects of neighborhood environments on young adult’s depressive mood [16], this study did not employ a multilevel model because the perceived neighborhood environment variables were measured at a personal level. The multilevel model assumes that some values are within more than two levels, but all variables in this research were measured at one personal level.

### 3.2. Data

We used KCHS data from 2019. These data were published by the Korea Center for Disease Control and Prevention (KCDC). All survey respondents were aged 19 or older. A stratified sampling was performed by the Dong/Eup/Myeon unit (a Korean administrative unit) and house type, and 900 people (error ±3%) were extracted by each community health center. In the statistics, the primary sampling point was determined in proportion to the household size by housing type, and the sample households were selected from the list of smaller sampling points using the systematic sampling method. The survey was executed by trained assistants using computer-assisted personal interviews (CAPI). The interviews were individual and conducted using laptops equipped with survey programs at the selected households. A total of 194,625 people over 35 and 34,474 people aged between 19 and 34 were surveyed in the 2019 KCHS.

### 3.3. Measured Variables

The measured variables and descriptive statistics are shown in Table 1 and Table 2 below. We divided all 24 independent variables into demographic and socioeconomic status factors, physical health and lifestyle habit factors, social network factors, social activity factors, and neighborhood environment factors. We selected these independent variables based on previous research results in our literature review. Our demographic and socioeconomic status factors included the following variables: gender, monthly household income, housing type, economic activity, single-person household, and marital status. Our physical health and lifestyle factors included the following variables: vigorous exercise, moderate exercise, walking, drinking level (high-risk drinking), daily life disturbance by the Internet, game and smartphone use, and stress. Our social network factors included the following variables: frequency of meeting families, meeting neighbors, and meeting friends (excluding neighbors). Our social activity factors included the following variables: religious activity, friendship activity, leisure activity, and volunteering activity. Our neighborhood factors included the following variables: SOC environment satisfaction, natural environment satisfaction, safety level satisfaction, trust between neighbors satisfaction, and public transportation environment satisfaction. In addition, as shown in Table 3, the VIF value of each dependent variable was below 10; thus, there was no multicollinearity between variables.

The dependent variable in this study was depressive mood as a continuous variable. We measured this variable via the Patient Health Questionnaire-9 (PHQ-9). The PHQ-9 contains nine question items and respondents can respond to each item via a four-point Likert-type scale, from 0 to 3. Their responses indicate the frequency of their depressive moods (where none = 0, several days in the last two weeks = 1, more than a week in the last two weeks = 2, and almost every day = 3). The PHQ-9 was originally used to assess depression symptoms and disorders. In this study, we used the PHQ-9 to measure depressive mood so that we could compare the degree of respondents’ depressive moods. We measured respondents’ depressive moods by summing their scores for each item (minimum = 0 points, maximum = 27 points). Among young adults in this study, 43.1% responded 0 on a feeling of depressive mood, with an average of 2.124 and a standard deviation of 3.112.

### 3.4. Analysis Model

A total of 40.8% (79,330 people) of the general adult group and 43.1% (14,931 people) of the young adult group had a PHQ-9 value of 0. When a part of the continuous dependent variable has a value 0 or less than 0, the Tobit model is suitable for analysis. The Tobit model can be used when some parts of dependent variables are 0, non-negative truncated values [57,58], although the original concept applied to the censored values. This model uses a latent continuous variable overall range of the models because the multiple regression has the dependent variable as a limited value [59]. The Tobit model Equation (1) uses the maximum likelihood method and assumes that the standard deviation follows the normal distribution:

(1) $$\begin{array}{c} y^{*}=\alpha +\beta x_{i}+e_{i},\;e_{i}\sim Ν\left(0,\;\sigma^{2}\right)\\ y_{i}=y_{i}^{*}\;\left(y^{*}>0\right),\;y_{i}=0\;\left(y^{*}\leq 0\right) \end{array}$$

The goodness of fit is measured using likelihood ratio (LR) test statistic, shown below in Equation (2). $L_{U}$ is the value of the likelihood function for the full model, which includes constant variables excluding the independent variables. $L_{R}$ is the value of the likelihood function of the restricted model, which contains all independent variables:

(2) $$\mathrm{LR}=-2\left(L_{R}-L_{U}\right)\sim \chi^{2}\left(df\right)$$

Because the Tobit model is a non-linear model, the coefficient that the model provides does not directly express the marginal effect. The influence of the independent variable is determined by the marginal effect of an independent variable on the probability of a dependent variable and the marginal effect on the truncated mean [60].

## 4. Results

### 4.1. The Goodness of Fit for the Tobit Model

The Tobit models were statistically significant for both the general adult group and the young adult group. As shown in Table 4, the LR(logliklihood ratio) the test statistics were 47,613.77 for the general adult group model and 8671.45 for the young adult group model in the chi-square distribution with df = 25. The probability of the LR(logliklihood ratio) test statistic in $\chi^{2}\left(25\right)$ was below 0.0000 in each model, which means that the models fit well.

### 4.2. Factors Affecting the Depressive Mood of Young Adults

Table 5 shows that the factors affecting depressive mood were identified at a significance level of 0.01 in both general and young adults. To compare the difference between the marginal effects of the two models, a two-tailed *t*-test was performed at a significance level of 0.01.

#### 4.2.1. Factors Affecting the Depressive Mood of Young Adults

In the young adult model, some variables affected depressive mood at a significance level of 0.01. The following variables were found to have an effect: gender, monthly household income, housing type, marital status, stress, drinking level (high-risk drinking), meeting neighbors, meeting friends (excluding neighbors), friendship activity, SOC environment satisfaction, natural environment satisfaction, safety level satisfaction, trust between neighbors satisfaction, and public transportation environment satisfaction. The following variables were found to have no effect: age, single-person household, vigorous exercise, moderate exercise, walking, meeting families, religious activity, leisure activity, and volunteering activity.

In terms of the demographic and socioeconomic status factors, women were 58.7% more depressed than men. People with low monthly household income experienced more depressive mood than people with relatively high monthly household income. People engaging in economic activity were 9.7% more depressed than people not engaging the economic activity. People living in apartments were 21.9% more depressed than people living in the other types of accommodation. People who did not have a spouse were 13.2% more likely to have depressive mood on average than those who had a spouse.

In terms of physical health and lifestyle habits, people with high stress experience the depressive mood 1.5 times more frequently on average than people with relatively low stress. Moreover, young adults with high-risk drinking behavior are 42.9% more likely to experience depressive mood than young adults without the high-risk drinking behavior. The more frequently that respondents met friends and families, the less depressed they were. In addition, their depressive mood was 4% lower when meeting families, and people who did activities with them more than once a month felt 29.8% less depressed than people who did not participate in activities with their friend at all.

#### 4.2.2. Factors Affecting the Depressive Mood of Young Adults Compared with General Adults

In the general adult model, all variables were significant except for vigorous exercise and leisure activity. The results of the t-test comparing general adults and young adults found that young adults’ depressive moods were more affected by gender, economic activity, stress, drinking level (high risk drinking), and meeting friends. In particular, the coefficients of economic activity were the opposite, and young adults felt more depressed when they were engaging in economic activity. More young adults (42.9%) engaged in heavy drinking than general adults (18.8%).

### 4.3. Relationship between Satisfaction with Neighborhood Environment and Young Adults’ Depressive Moods

Table 6 describes the correlation of each neighborhood environment factor. The correlation between the three variables of SOC environment, natural environment, and safety level was 0.376 on average, but not enough to be treated as one variable.

**Figure 1:**
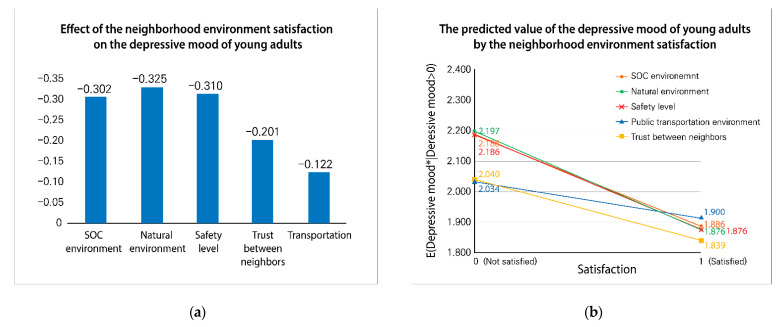
(**a**) Effects of neighborhood environment satisfaction on depressive mood; (**b**) The predictive value of the depressive mood of young adults by the neighborhood environment satisfaction.

Table 6 shows that all five neighborhood environment variables had a significant effect on the depressive mood of young adults. Table 5 shows the marginal effect of these variables. The marginal effect reveals the impact magnitude of an independent variable statistically, which is the change of the dependent variable when one unit of the independent variable changes. The marginal effects of neighborhood environments were the natural environment (32.5%), safety level (31.0%), SOC environment (30.2%), trust between neighbors (20.1%), and public transportation environment (12.2%) (see Figure 1a). Figure 1b identifies the predicted values of SOC environment satisfaction, natural environment satisfaction, and safety level satisfaction (for dissatisfaction/satisfaction, SOC environment 2.188/1.872, natural environment 2.197/1.872, and for safety level 2.186/1.876). When unsatisfied, the predicted value of trust between neighbor satisfaction (2.040) was similar to that of the public transportation environment satisfaction (2.034). Regarding satisfaction, the predicted value of trust between neighbor satisfaction (1.839) was lower than that of public transportation environment satisfaction (1.912). Therefore, it was found that satisfaction with trust between neighbors was more effective than satisfaction with public transportation environment in lowering depressive moods.

## 5. Discussion

### 5.1. Corroboration with Previous Studies

#### 5.1.1. General Factors Affecting Young Adults’ Depressive Mood

In this study, we found significant differences in the factors affecting young adults’ depressive mood compared to the general adults’ depressive mood. In particular, the effect of economic activity and meeting friends (excluding neighbors) were very significant compared to previous studies. Differences in gender, stress, and drinking level, which were consistent with most previous studies, had stronger effects than other factors.

Drinking level is known to considerably influence depression [41]. This study found that drinking level factors were 2.3 times more influential for young adults than they were for general adults—i.e., that young adults were more affected by depressive mood when they were in high-risk drinking status. Young adults tend to engage in dangerous behaviors such as drinking and smoking. Considering their characteristics, young adults would be more exposed to depressive mood by high-risk alcohol experiences than other adults.

Regarding economic activity, there could be another influential factor for young adults’ work apart from unemployment. Young adults had a higher depressive mood when engaged in economic activity (0.097), and general adults had a lower depressive mood when engaged in economic activity (–0.437). Previous research from South Korea has found that unemployment causes depression in young adults [9], in addition to this, this study found that work-related stress can influence young adults’ depressive mood while unemployment still causes depression.

Regarding social support, meeting friends had a higher effect on the depressive mood of young adults (9%) than on general adults (5%). Our findings regarding young adults run counter to some studies [46] which concluded that meeting with friends does not influence young adults’ depressive moods but are corroborated by other studies on young adults in South Korea have indicated that support from friends is as important as support from family [61]. Our findings regarding the general adult population and the effects of meeting friends are supported by previous studies done by Western researchers [44]. We also found that differences in physical activity did not influence young adults’ depressive mood at a statistically significant level. This finding implied that while depressive moods is driven by physical activity (or a lack thereof) among the elderly, young adults’ depressive moods is driven by cognitive factors.

#### 5.1.2. Neighborhood Environment Factors and Their Effects on Young Adults’ Depressive Moods

Our analysis indicated that a neighborhood’s natural environment, (32.5%), safety level (31.0%), and SOC environment (30.2%) had a similar effect on young adults’ depressive moods as their friendship activities (29.8%). As shown in Table 6 and mentioned above, the correlation values of these three variables indicate that many young adults are unsatisfied by all three elements of their neighborhood environments (natural environment, safety level, and SOC environment) simultaneously. Our finding that all three of these elements contribute to young adults’ depressive mood is corroborated by various other studies [15,17,51]. Perhaps most importantly, we found that the effects of young adults’ satisfaction with their SOC environment (e.g., electricity, water and sewage, garbage collection, sports facilities, etc.) on their depressive moods indicates that the functional aspects of a neighborhood environment can contribute to depressive moods. Because other studies have found that hassles in daily life can affect people’s depressive mood [62], we suggest that poor-quality SOC facilities could worsen mental health. 

Our study also found that young adults felt 20.1% more depressed when they did not trust their neighbors. Trust between neighbors is known to influence depressive mood [63], therefore, although the effect of this factor was lower than the three elements of neighborhood environments described above, it is still a contributing factor. It is understood that the effect degree of trust between neighbors was small because of the characteristics that the social relations of young adults were mainly formed outside the neighborhood.

Our study also found that young adults’ satisfaction with the public transportation environment had the smallest effect on their depressive mood (12.2%). Even when some were satisfied with their neighborhood public transportation environment, the predicted value of depressive mood was the highest at 1.192. In other words, young adults who were satisfied with public transportation environment were more depressed than young adults who were satisfied with the other neighborhood environments. Other studies have shown that Korean young adults value the access to public transportation as the most important factor when choosing housing [64]. This means that they are, as a group, sensitive to their neighborhood public transportation environments. A few studies have found that differences in elderly people’s satisfaction with their local public transportation environment affects their depressive moods as well [65,66,67]. Although the marginal effect of public transportation environment satisfaction is not as large as that of other neighborhood environment factors among young adults, we identify it here as a factor that influences their depressive mood.

### 5.2. Limitations and Strengths

This study makes several strong contributions to the literature. For example, it identifies specific causes of young adults’ depressive moods in their neighborhood environment—namely, their satisfaction with their neighborhood’s natural environment, safety level, SOC environment, trust between neighbors, and public transportation environment. In particular, the results regarding the effect of the SOC environment contributes to the literature.

This study has a few limitations, however. For example, we measured neighborhood environment satisfaction variables using dummy variables, consisting of 0 and 1, in national statistics; therefore, our study cannot make detailed analyses. In addition, this study only identified that there is indeed a relationship between the neighborhood environment and depressive mood. Future research is should attempt to explain the relationship between neighborhood environment and depressive moods factors in more detail. 

In addition, the severity of depression was measured with the PHG-9 and a self-rating scale, instead of using a clinical rated scale such as the Hamilton Rating Scale for Depression [68,69]. Therefore, the results cannot be interpreted clinically, and the depressive mood needs to be understood in comparison to the non-clinical depressive mood.

## 6. Conclusions

Many modern South Koreans suffer from depression and other psychological burdens as a result of contemporary crises in housing, the national economy, and so on. Although the South Korean government has provided some financial support to young adults in response to this growing problem, many young adults still live in an inadequate neighborhood environment. This study used the Tobit model to analyze KCHS data from 2019 and better understand the effects of the neighborhood environment on young adults’ depressive moods. This study: (1) analyzed the factors affecting young adults’ depressive moods by comparing their results with those of adults over 35; (2) confirmed the effects of perceived neighborhood environments on depressive mood reported in previous literature; and (3) compared the magnitude of each effect related to the neighborhood environment on the depressive mood of young adults.

It was found that the following variables affect young adults’ depressive moods: gender, monthly household income, economic activity, housing type, marital status, stress, drinking level, meeting friends, meeting neighbors, and friendship activities. It was also found that young adults’ satisfaction with their natural environment, safety level, SOC environment, trust between neighbors, and public transportation environment in neighborhood scale all affected young adults’ depressive moods. Each of these neighborhood environment satisfaction factors affected their depressive moods. Satisfaction with neighborhood safety level had the largest effect, followed by satisfaction with their SOC environment, trust between neighbors, and public transportation environment.

This research contributes to the literature by identifying how young adults’ neighborhood environment causes their depressive moods. The results can therefore help policymakers design mental health and environmental initiatives for young adults and perhaps design urban environments that can help people sustain good mental health.

## Figures and Tables

**Table 1:** Measured variables.

Type of Variable	Category	Variable	Measurement Unit
Dependent variable	Depressive mood		Patient Health Questionnaire-9(PHQ-9) scores
Independent variable	Demographic and socioeconomic status factors	Age	Age
		Gender	Man = 0, woman = 1
		Monthly household income	Less than $500(USD) = 1, more than $500~less than 1000 = 2, more than $1000~less than 2000 = 3, more than $2000~less than 3000 = 4, more than $3000~less than 4000 = 5, more than $4000~less than 5000 = 6, more than $5000~less than 6000 = 7, more than $6000 = 8
		Economic activity	No = 0, yes = 1 Whether respondent is engaged in economic activity
		Housing type	The other types = 0, apartment = 1
		Single-person household	Not single-person household = 0, single-person household = 1
		Marital status	Having spouse = 0, unmarried = 1, divorce/bereavement/separation = 2
	Physical health and lifestyle habits	Vigorous exercise	No = 0, yes = 1 Practicing vigorous exercise more than 20 min a time, 3 times a week
		Moderate exercise	No = 0, yes = 1 Practicing moderate exercise more than 30 min a time, 5 times a week
		Walking	No = 0, yes = 1 Walking more than 30 min a time, 5 times a week
		Stress	Normal = 0, mild = 1, moderate = 2, severe = 3
		Drinking level(High-risk drinking)	Not high-risk drinking = 0, high-risk drinking = 1 High-risk drinking: more than seven cups of Soju for men, more than five cups of Soju for women, more than twice a week
	Social network	Meeting family	Less than once a month = 0, once a month = 1, 2~3 times a month = 2, once a week = 3, 2~3 times a week = 4, more than 4 times a week = 5
		Meeting neighbors	
		Meeting friends (excluding neighbors)	
	Social activity	Religious activity	No = 0, yes = 1 Regular religious activity more than once a month
		Friendship activity	No = 0, yes = 1 Regular friendship activity more than once a month
		Leisure activity	No = 0, yes = 1 Regular Leisure activity more than once a month
		Volunteering activity	No = 0, yes = 1 Regular volunteering activity more than once a month
	Neighborhood environment satisfaction	SOC environment satisfaction	Not satisfied = 0, satisfied = 1 Satisfaction with the neighborhood’s living environment (electricity, water and sewage, garbage collection, sports facilities, etc.)
		Natural environment satisfaction	Not satisfied = 0, satisfied = 1 Satisfaction with the neighborhood’s natural environment (air quality, water quality, etc.)
		Safety level satisfaction	Not satisfied = 0, satisfied = 1 Satisfaction with the overall safety level of the neighborhood (natural disaster, traffic accident, farm work accident, crime, etc.)
		Trust between neighbors satisfaction	Not satisfied = 0, satisfied = 1 Neighbors can trust each other
		Public transportation environment satisfaction	Not satisfied = 0, satisfied = 1 Satisfaction with the neighborhood traffic conditions (bus, taxi, subway, train, etc.)

**Table 2:** Descriptive statistics.

Type of Variable	Category	Variable	General Adults Group (35+)		Young Adults Group (19–34)	
			Avg.	S.D.	Avg.	S.D.
Dependent variable	Depressive mood		2.124	3.095	2.124	3.114
Independent variable	Demographic and socioeconomic status factors	Age	59.993	13.903	26.649	4.607
		Gender	0.558	0.497	0.520	0.500
		Monthly household income	4.693	2.118	5.867	1.808
		Economic activity	0.619	0.486	0.623	0.485
		Housing type	0.387	0.487	0.555	0.497
		Single-person household	0.169	0.374	0.109	0.312
		Marital status	0.478	0.821	0.745	0.453
	Physical health and lifestyle habits	Vigorous exercise	0.131	0.338	0.193	0.395
		Moderate exercise	0.140	0.347	0.123	0.328
		Walking	0.388	0.487	0.480	0.500
		Stress	0.973	0.743	1.179	0.718
		Drinking level (High-risk drinking)	0.108	0.311	0.133	0.340
	Social network	Meeting families	2.981	1.791	2.545	1.953
		Meeting neighbors	2.894	2.092	1.122	1.741
		Meeting friends (excluding neighbors)	2.564	1.893	3.451	1.641
	Social activity	Religious activity	0.291	0.454	0.164	0.370
		Friendship activity	0.578	0.494	0.367	0.482
		Leisure activity	0.269	0.443	0.331	0.471
		Volunteering activity	0.091	0.287	0.035	0.183
	Neighborhood environment satisfaction	SOC environment satisfaction	0.851	0.356	0.778	0.416
		Natural environment satisfaction	0.824	0.381	0.747	0.435
		Safety level satisfaction	0.856	0.352	0.750	0.433
		Trust between neighbors satisfaction	0.693	0.461	0.435	0.496
		Public transportation environment satisfaction	0.720	0.449	0.676	0.468

**Table 3:** Variance Inflation Factor (VIF) between independent variables.

Variable	VIF	1/VIF
Age	1.65	0.604901
Gender	1.61	0.621311
Monthly household income	1.36	0.737471
Economic Activity	1.32	0.755001
Housing type	1.29	0.77376
Single-person household	1.28	0.77945
Marital status	1.25	0.79798
Vigorous exercise	1.22	0.821449
Moderate exercise	1.2	0.834955
Walking	1.2	0.836534
Stress	1.19	0.84335
Drinking level (High-risk drinking)	1.17	0.856265
Meeting families	1.16	0.858939
Meeting neighbors	1.16	0.864411
Meeting friends (excluding neighbors)	1.15	0.872968
Religious activity	1.11	0.898413
Friendship activity	1.11	0.901332
Leisure activity	1.09	0.9191
Volunteering activity	1.08	0.923754
SOC environment satisfaction	1.06	0.939281
Natural environment satisfaction	1.05	0.951834
Safety level satisfaction	1.05	0.954549
Trust between neighbors satisfaction	1.04	0.963885
Public transportation environment satisfaction	1.03	0.969137
Mean VIF	1.2	

**Table 4:** Goodness of fit for the Tobit model.

Goodness of Fit	Tobit Model for the General Adult Group	Tobit Model for the Young Adult Group
$LR\left(logliklihood\;ratio\right)\sim \chi^{2}\left(25\right)$	47,613.77	8671.45
$Probability\;of\;LR\;test\;statistic\;\chi^{2}\left(25\right)$	0.0000 *	0.0000 *

* Numbers below five decimal places could not be seen with our statistical tools/software.

**Table 5:** Marginal effects in the Tobit model: comparing the general adult and young adult models.

Variable		General Adults Model (35+)			Young Adults Model (19–34)			Difference	
		dy/dx	SE	*p*-Value	dy/dx	SE	*p*-Value	|t|	$|t|-z_{0.005}$
Demographic and socioeconomic status factors	Age	0.028 **	0.001	0.000	0.005	0.004	0.224	6.133	2.408 **
	Gender *	0.440 **	0.013	0.000	0.587 **	0.028	0.000	4.742	1.017 **
	Monthly household income	−0.096 **	0.004	0.000	−0.070 **	0.008	0.000	2.941	−0.784
	Economic activity *	−0.437 **	0.014	0.000	0.097 **	0.031	0.001	15.973	12.248 **
	Housing type *	0.128 **	0.013	0.001	0.219 **	0.029	0.000	2.874	−0.851
	Single-person household *	0.069 **	0.020	0.000	0.106	0.050	0.033	0.690	−3.035
	Marital status	0.193 **	0.009	0.010	0.132 **	0.037	0.000	1.599	−2.126
Physical health and lifestyle habits	Vigorous exercise *	0.046	0.018	0.724	0.056	0.037	0.130	0.244	–3.481
	Moderate exercise *	0.006 **	0.017	0.000	0.022	0.043	0.615	0.338	−3.387
	Walking *	–0.192 **	0.012	0.000	−0.005	0.028	0.857	6.197	2.472 **
	Stress	1.350 **	0.008	0.000	1.520 **	0.020	0.000	7.999	4.274 **
	Drinking level (high-risk drinking) *	0.188 **	0.020	0.000	0.429 **	0.044	0.000	5.008	1.283 **
Social network	Meeting families	−0.026 **	0.003	0.000	0.025	0.007	0.001	6.315	2.590 **
	Meeting neighbors	−0.045 **	0.003	0.000	−0.040 **	0.008	0.000	0.606	−3.119
	Meeting friends (excluding neighbors)	−0.052 **	0.003	0.000	−0.099 **	0.009	0.000	4.986	1.261 **
Social activity	Religious activity *	−0.047 **	0.013	0.000	−0.002	0.037	0.963	1.149	−2.576
	Friendship activity *	−0.331 **	0.013	0.000	−0.298 **	0.030	0.000	1.026	−2.699
	Leisure activity *	−0.102	0.014	0.256	0.080	0.032	0.012	5.222	1.497 **
	Volunteering activity *	−0.024 **	0.021	0.000	−0.007	0.076	0.923	0.210	−3.515
Neighborhood environment satisfaction	SOC environment satisfaction *	−0.194 **	0.018	0.000	−0.302 **	0.038	0.000	2.542	−1.183
	Natural environment satisfaction *	−0.312 **	0.017	0.000	−0.325 **	0.036	0.000	0.321	−3.404
	Safety level satisfaction *	−0.262 **	0.019	0.000	−0.310 **	0.037	0.000	1.150	−2.575
	Trust between neighbors satisfaction *	−0.225 **	0.014	0.000	−0.201 **	0.029	0.000	0.759	−2.966
	Public transportation environment satisfaction *	−0.203 **	0.013	0.000	−0.122 **	0.030	0.000	2.470	−1.255

* Dummy variable (consist of 0, 1); ** Statistically significant at the level of 0.01.

**Table 6:** Correlations between the sectors of neighborhood environment: Young adult group (19–34).

Satisfaction Section	SOC Environment	Natural Environment	Safety Level	Trust between Neighbors	Public Transportation Environment
SOC environment	1.000	0.362	0.377	0.172	0.229
Natural environment	0.362	1.000	0.388	0.205	0.060
Safety level	0.377	0.388	1.000	0.287	0.133
Trust between neighbors	0.172	0.205	0.287	1.000	0.060
Public transportation environment	0.229	0.060	0.133	0.060	1.000

## Data Availability

The data presented in this study are available on request from the Korea Disease Control and Prevention Agency (KDCA). The request for data can be found here: https://chs.cdc.go.kr/chs/rdr/rdrInfoPledgeMain.do#.
